# Isolation and Characterization of Acetylated Derivative of Recombinant Insulin Lispro Produced in *Escherichia coli*

**DOI:** 10.1007/s11095-015-1637-y

**Published:** 2015-02-07

**Authors:** Joanna Szewczak, Anna Bierczyńska-Krzysik, Marcin Piejko, Paweł Mak, Dorota Stadnik

**Affiliations:** 1 Institute of Biotechnology and Antibiotics, Starościńska 5, 02-516 Warsaw, Poland; 2 Department of Analytical Biochemistry, Faculty of Biochemistry, Biophysics and Biotechnology, Jagiellonian University, Gronostajowa 7, 30-387 Kraków, Poland; 3 Jagiellonian University Medical College, Św. Anny 12, 31-008 Kraków, Poland; 4 Malopolska Centre of Biotechnology, Jagiellonian University, Gronostajowa 7, 30-387 Kraków, Poland

**Keywords:** Edman degradation, lysine acetylation, peptide mapping, posttranslational modifications, recombinant proteins

## Abstract

**Purpose:**

Insulin lispro is a rapid-acting insulin analogue produced by recombinant DNA technology. As a biosynthetic drug, the protein undergoes strict monitoring aiming for detection and characterization of impurities. The goal of this study was to isolate and identify a derivative of insulin lispro formed during biosynthesis.

**Methods:**

For this purpose, ion exchange chromatography in combination with endoproteinase Glu-C digestion, MALDI-TOF/TOF mass spectrometry and Edman sequencing were employed.

**Results:**

Ion exchange chromatography analysis of related proteins in development batches of recombinant insulin lispro revealed the existence of unknown derivative in excess of the assumed limit. Its molecular mass was 42 Da higher than the theoretical mass of Lys(B31) insulin lispro—one of the expected process-related intermediates. Endoproteinase Glu-C cleavage enabled indication of the modified peptide. Tandem mass spectrometry (MS/MS) allowed to explore the location and type of the modification. The 42 amu shift was present in the mass of y-type ions, while b-type ions were in agreement with theoretical values. It suggested that the modification is present on B31 lysine. Further inquiry revealed the presence of two diagnostic ions for lysine acetylation at m/z 143.1 and 126.1. In addition, the peptide was isolated and sequenced by Edman degradation. Standards of phenylthiohydantoin derivatives of N-ε-acetyl-L-lysine and N-ε-trimethyl-L-lysine, not available commercially, were synthesized in the laboratory. The retention time of the modified residue confirmed its identity as N-ε-acetyl-L-lysine.

**Conclusions:**

The derivative of insulin lispro formed during biosynthesis of the drug was identified to be N-ε-acetyl-L-lysine (B31) insulin lispro.

## INTRODUCTION

In 1980s recombinant human insulin has been implemented as a base treatment for patients suffering from diabetes (1). However, when administered, the drug did not mimic physiological, 24-h long profile of endogenous hormone release by beta-cells. Therefore, in recent years, analogues of human insulin have been engineered to improve insulin therapy through modulation of onset and duration of action after injection. Insulin lispro is a rapid-acting insulin analogue designed by inversion of two penultimate amino acids: proline (B28) and lysine (B29) on the C-terminal end of the B-chain of human insulin. The subtle change in the amino acid sequence does not alter receptor binding, whereas it considerably diminishes the insulin’s natural propensity to self-associate to dimers and higher order oligomers (2). Larger amounts of active monomeric insulin available for postprandial injections enable significantly faster glucose level reduction. As a result, the insulin analogue shortens noticeably the time interval between the injection and a meal, offering a greater flexibility with meal times (3).

The analogue is produced by recombinant DNA technology in *E. coli* as a fusion protein which is further converted to the functional hormone by selected enzymes. However, inherent feature of all recombinant proteins is the presence of post-translational modifications (PTMs) related to their production system and storage (4–6). All PTMs present in a protein drug product may potentially affect its safety and efficacy and should be evaluated in terms of toxicity, immunogenicity and biological activity. In living cells PTMs result in structural and functional heterogeneity and thus can affect numerous biological processes *e.g.* DNA replication and repair, cellular signalling, cell growth, metabolism, development and apoptosis (7–9). In broader perspective, understanding of biological implications of PTMs on human health is of significant value as it is still unclear if PTMs are the cause or the consequence of certain diseases (10). One such PTM is lysine acetylation which is gaining increasing attention. The recent reports concerning many thousands of acetylated proteins in both eukaryotes and prokaryotes suggest that lysine acetylation is involved in regulation of diverse substantial cellular processes such as chromatin remodeling, protein synthesis, nuclear transport, cell cycle and the coordination of different metabolic pathways (11–14).

It was previously acknowledged that lysine acetylation was regulated by enzymes such as lysine acetyltransferases (KATs) and deacetylases (KDACs) (15). However, B. N. Violand *et al.* suggested that acetylation of lysines may occur by chemical mechanism with acetyl-CoA or another metabolic intermediate providing the source of the acetyl group (16). Indeed, recent studies have shown that lysine acetylation can also proceed non-enzymatically using acetyl-CoA (17) and acetyl phosphate (AcP) as the acetyl donor (18). Interestingly, an analogous AcP-dependent non-enzymatic reaction was observed earlier for phosphorylation (19,20). AcP is a small-molecule metabolite in the phosphotransacetylase (Pta)—acetate kinase (AckA) pathway, one of the basic metabolic networks in *E. coli* (19,21). In light of the recent explosion of basic research on lysine acetylation (22–24), it seems important to recall previous studies on acetylation of recombinant therapeutic proteins produced in *E. coli*: neurotropin-3 (25), somatotropin (16), interleukin-10 (26), interleukin-2 (27), chemokine RANTES (regulated on activation, normal T cell expressed and secreted) (17), interferon alfa (28) and gamma (29), stathmin-like subdomains (30), human basic fibroblast growth factor mutein (31) and prochymosin (32). These results support current attempts to understand the mechanism of acetylation and its implications on cell life. Moreover, new discoveries should be expected in this filed since *E. coli* is still one of the most widely used expression hosts in the biopharmaceutical industry. Nearly 30% of currently approved recombinant therapeutic proteins are produced in this system (33). In concern for patients, the drug approval by regulatory authorities is dependent on still growing demands related to its efficacy, quality and safety (34). Impurities and derivatives of therapeutic proteins should be removed in a purification step or, if present in acceptable quantities, characterized to the greatest possible extent.

**Fig. 1:**
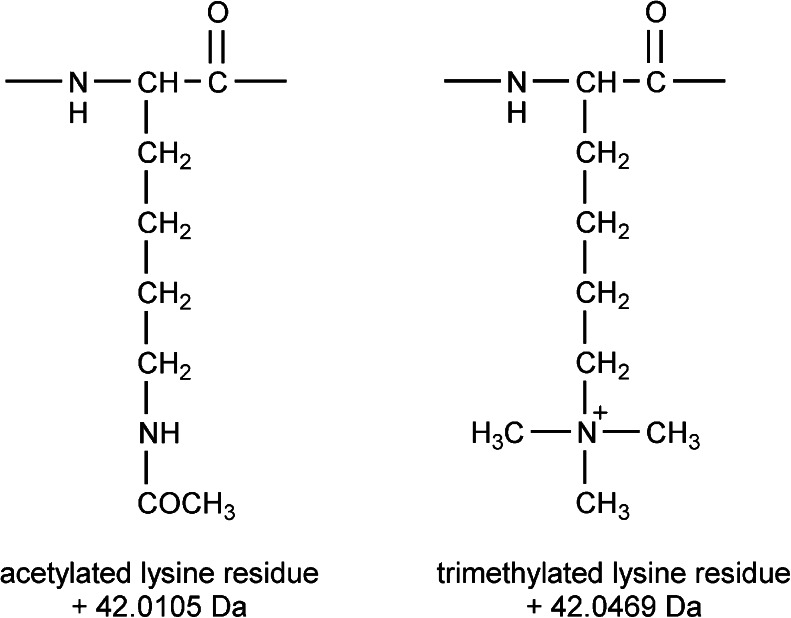
Structures of acetylated and trimethylated lysine residues.

The purpose of this work was to isolate and identify an acetylated derivative formed during biosynthesis of insulin lispro. The main difficulty in characterization of the derivative was its limited availability. Moreover, a mass coincidence between acetylated and tri-methylated protein derivatives (see Fig. 1) was a challenge for mass spectrometry analysis.

We applied ion exchange chromatography to isolate the derivative from insulin lispro substance. The derivative was further characterized by combination of enzymatic digestion, mass spectrometry and Edman sequencing. The results confirmed interchangeably that the protein isolated from a production batch is an acetylated derivative of recombinant lispro insulin.

## MATERIALS AND METHODS

### Source of Insulin Lispro

Insulin lispro was produced by recombinant DNA technology essentially as described previously (35). Shortly, insulin lispro precursor was expressed in *E. coli* in the form of insoluble inclusion bodies. After separation from cell debris, the precursor was transformed into insulin lispro by enzymatic digestion with trypsin and carboxypeptidase B. Finally, insulin was purified by chromatography, dialysis and crystallization.

### Isolation of the Insulin Lispro Acetylated Derivative

The derivative was isolated from a development batch of insulin lispro substance by repetitive ion exchange chromatography (IEC) using ICS-5000 system with a PDA detector (Dionex, Sunnyvale, USA). 0.1 mg of the insulin lispro containing 0.8% of the derivative was injected on a DNA Pac PA-100, 250 × 4.0 column (Dionex, Sunnyvale, USA). The separation was carried out at 35°C with a linear gradient elution from 0% to 25% eluent B in 30 min at the flow rate 0.5 ml/min. Eluent A was 8 mM phosphate buffer, 67% ethanol pH 7.6 and eluent B was 0.3 M NaCl, 8 mM phosphate buffer, 67% ethanol pH 7.6. The peak of the derivative (relative retention time 0.8; see Fig. 3) was collected. This procedure was repeated several times to obtain sufficient amount of the derivative. All fractions of the derivative were pooled and evaporated to dryness using Concentrator Plus vacuum centrifuge (Eppendorf, Hamburg, Germany) and kept at −20°C until use.

### Peptide Mapping and Isolation of Peptide B22-B31

The derivative was dissolved in 0.5 ml of 0.1 M HEPES buffer pH 7.5 and mixed with 10 μl of 10 μg/ml endoproteinase Glu-C (Protease *S. aureus* V8; Thermo Scientific, Rockford, USA). The mixture was incubated for 4 h at 37°C. Then the HPLC system (Alliance 2695, Waters, Milford, USA) with UV detector (2489, Waters, Milford, USA), equipped with a Zorbax SB-C18 1.8, 4.6 × 50 mm column (Agilent, Palo Alto, USA), was employed to separate and isolate the peptides. The separation was carried out at 40°C with a linear gradient elution from 10% to 50% eluent B in 20 min at a flow rate 1 ml/min. Eluent A was 0.1% trifluoroacetic acid (TFA) and eluent B was 0.1% TFA with 90% acetonitrile (ACN) (both vol./vol.). The peak of the peptide B21-B31 was collected with Fraction Collector III (Waters, Milford, USA) and eluent was evaporated as mentioned above.

### Synthesis of Phenylthiohydantoin Amino Acid Standards for Protein Sequencing

The water solutions of N-ε-acetyl-L-lysine (acK) and N-ε-N-ε-N-ε-trimethyl-L-lysine (3meK, both from Sigma, St. Louis, USA), containing 50 nmol each, were evaporated to dryness in Eppendorf tubes and converted into phenylthiocarbamyl (PTC) derivatives essentially as described elsewhere (36). Obtained dry deposits were then converted into phenylthiohydantoin (PTH) derivatives by dissolving them in 100 μl portions of 25% (vol./vol.) TFA, 0.001% (wt./vol.) dithiothreitol in water, flushing with argon, sealing, and incubation for 30 min at 64°C. After this step the PTH derivatives were purified by HPLC on a LC-18-DB 4.6 × 250 mm column (Supelco, Bellefonte, USA). Two solvents were used: A – 0.1% TFA (all vol./vol.) and B – 0.07% TFA, 80% ACN (vol./vol.). The linear gradient 0–100% of the solvent B over 15 min, spectrophotometric detection at 269 nm, and 1 ml/min flow rate were applied. Fractions containing PTH-acK and PTH-3meK were collected, evaporated to dryness, and kept at −20°C until use.

### N-terminal Sequencing

Determination of N-terminal sequence of polypeptide chains by Edman degradation was performed on a PPSQ30 (Shimadzu, Kyoto, Japan) automatic protein sequencer. Before analyses the instrument was calibrated using a commercial PTH proteinaceous amino acid standard mixture (Wako, Osaka, Japan) and, separately, using the synthesized PTH-acK as well as PTH-3meK. The calibration was performed by 25 pmol amounts of these compounds, dissolved previously in 37% (vol./vol.) ACN in water. The sequencing was done using polypeptide chains absorbed on TFA-treated glass fiber disks (Wako, Osaka, Japan) coated by polybrene (Sigma, St. Louis, USA).

### Mass Spectrometry

The insulin lispro derivative and endoproteinase Glu-C derived peptides were analyzed in a reflector mode with the use of 4800 Plus MALDI-TOF/TOF mass spectrometer (AB SCIEX, Framingham, USA). α-cyano-4-hydroxy-cinnamic acid matrix was exploited. External calibration was performed with a 4700 proteomics analyzer calibration mixture provided by AB SCIEX. Data Explorer Software, Version 4.9, and General Protein/Mass Analysis for Windows, version 8.2, were applied for assignment of a, b and y ions to the MS/MS spectrum of acetylated peptide. DeNovo Explorer (GPS Explorer TM Software, Version 3.6) was exploited to match the MS/MS spectrum to the annotated peptide sequence.

## RESULTS AND DISCUSSION

**Fig. 2:**
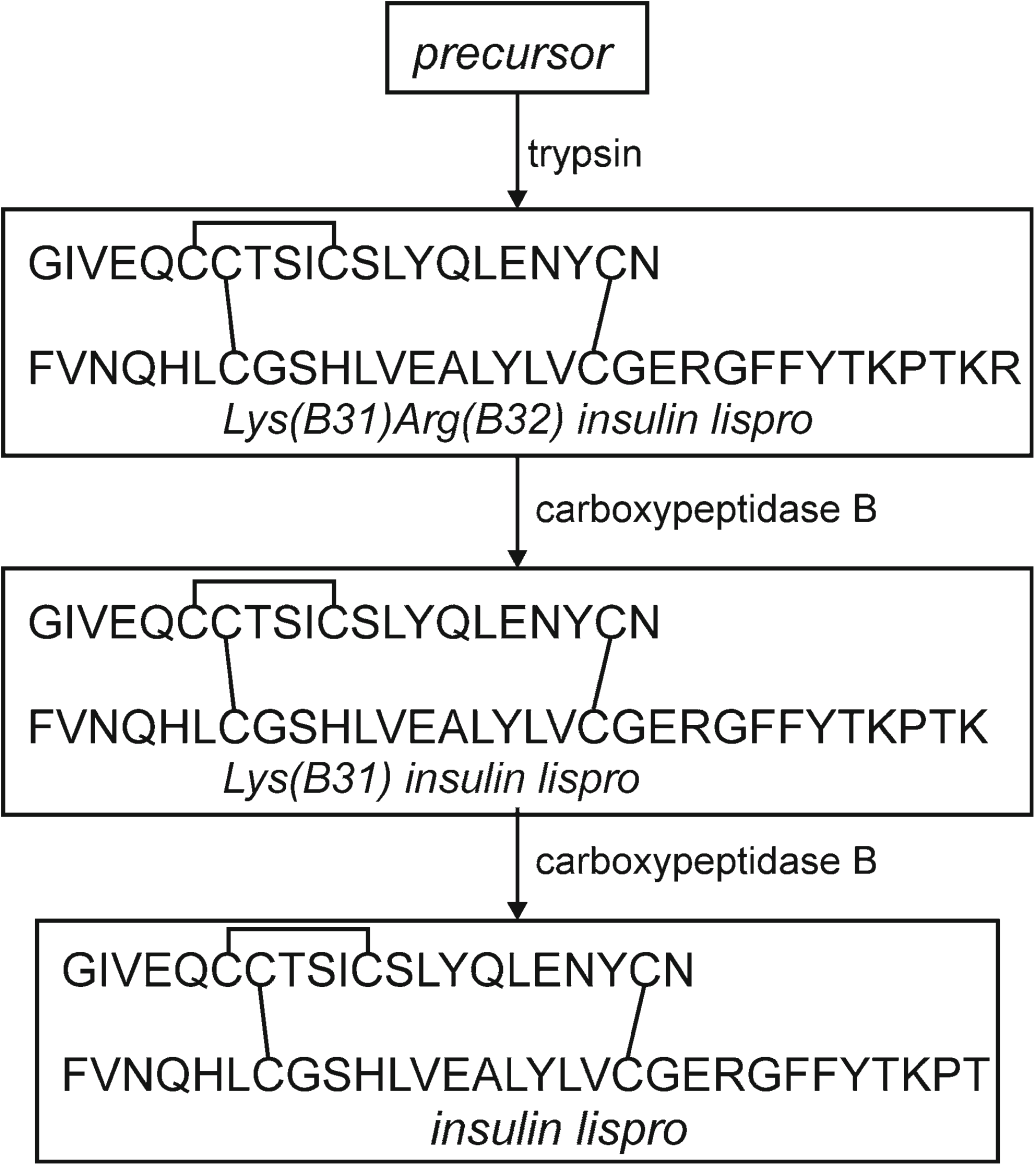
Scheme of enzymatic conversion of precursor during the production process of insulin lispro.

Insulin lispro is a short-acting insulin analogue produced by recombinant DNA technology in *E. coli* as a precursor which is further converted to the native hormone by enzymes (Fig. 2).

**Fig. 3:**
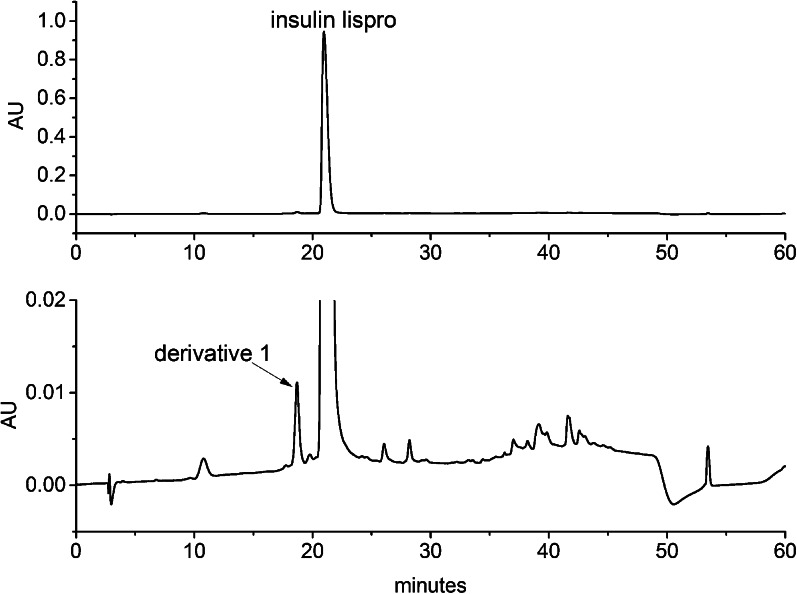
IEC chromatogram of insulin lispro.

During the production process, different impurities called related proteins are formed at both biosynthesis and purification steps. Related proteins constitute various modifications of a desired protein including deamidated, methylated, oxidized, cleaved, aggregated forms and adducts, just to name a few, and they are present in a final bulk of a drug substance. All can be monitored by various chromatographic methods. Figure 3 illustrates the IEC-chromatogram of insulin lispro manufactured at Institute of Biotechnology and Antibiotics in Warsaw.

**Fig. 4:**
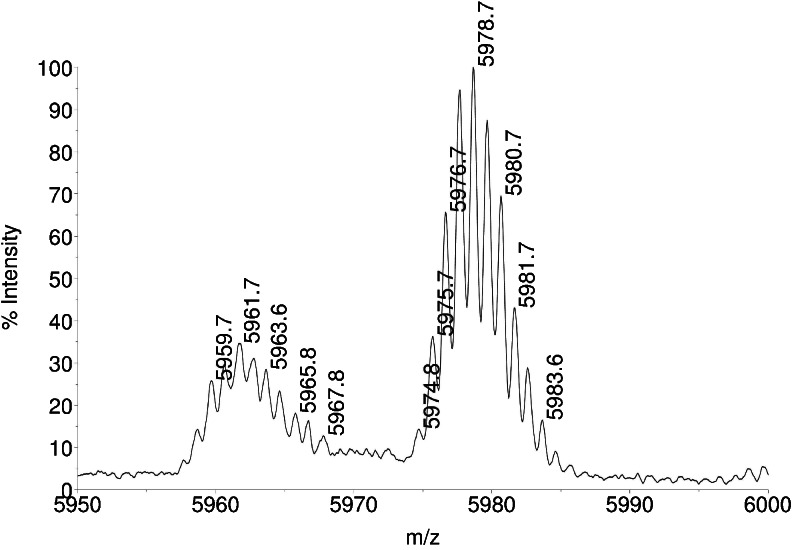
MALDI TOF spectrum of derivative 1.

The main peak in this chromatogram is insulin lispro. Small peaks arising from the baseline (seen in enlargement in Fig. 3) are related proteins. The derivative peak 1 was isolated and subjected for further investigation. Taking into account the retention time relative to insulin lispro, derivative 1 was suspected to be one of intermediates possessing additional basic amino acids at chain B: Lys(B31) insulin lispro or Lys(B31)Arg(B32) insulin lispro, which can appear during enzymatic conversion of the precursor (see Fig. 2). Surprisingly, MALDI analysis of the collected fraction of peak 1 showed a +42 mass increment when compared to the theoretical mass of Lys(B31) insulin lispro (5932.7 Da) (Fig. 4). Such a change suggests trimethylation or acetylation of lysine. On the basis of the recent discoveries of serine and threonine acetylation by a bacterial effector from *Yersinia* (37), these residues were also considered as suspicious during our study. Moreover, two modifications, methylation and formylation, can occur simultaneously within Lys(B31) insulin lispro molecule resulting in +42 mass shift (14 Da + 28 Da).

**Fig. 5:**
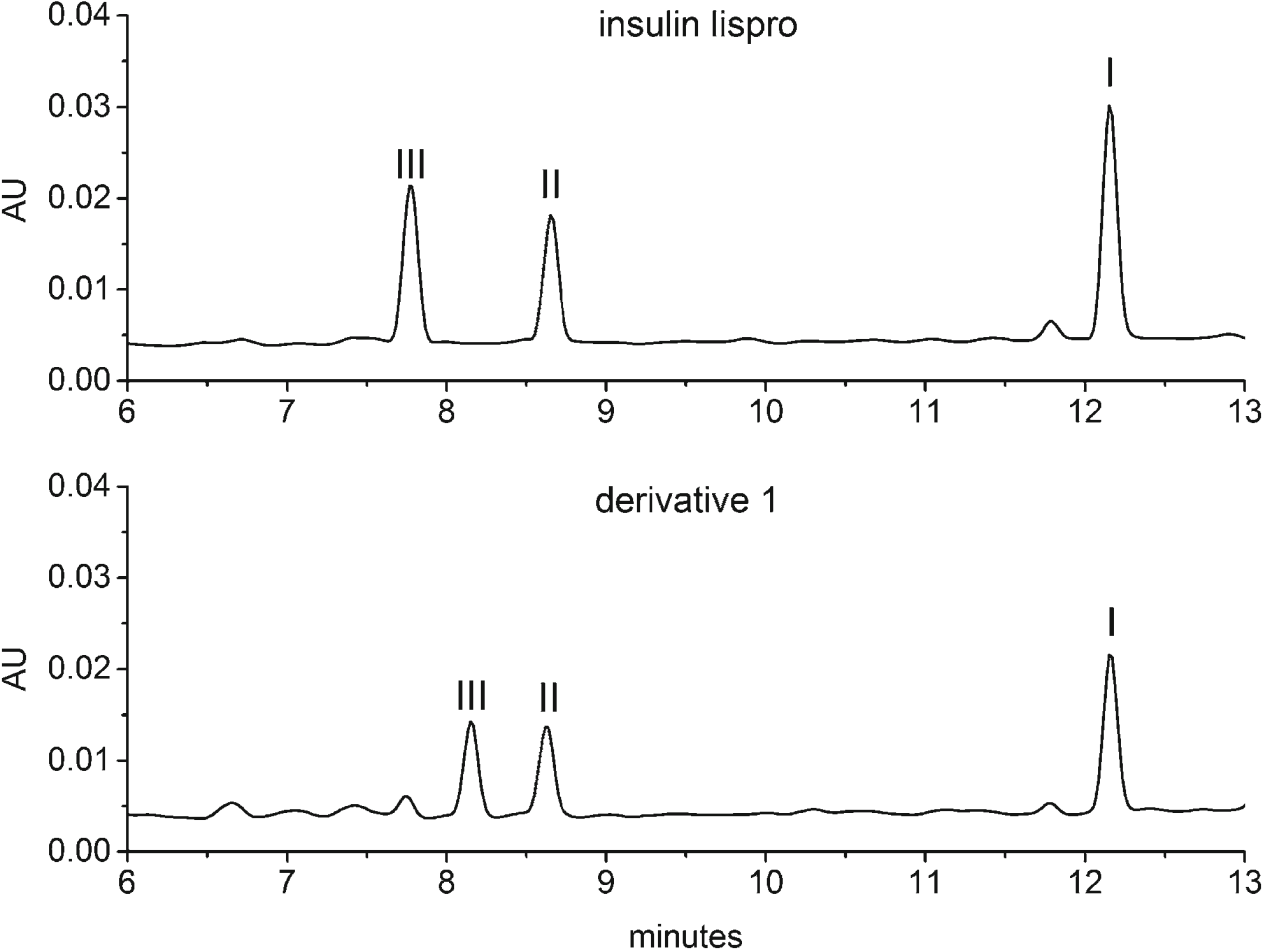
Peptide maps (RP-HPLC chromatograms) of insulin lispro and the derivative 1 after enzymatic digestion with endoproteinase Glu-C; fragment IV eluted at dead time.

Enzymatic digestion and peptide mapping were employed to identify the modification site of the derivative. The experiment was performed in parallel with insulin lispro. The complete digestion of insulin lispro with endoproteinase Glu-C results in four fragments: I (A5-A17, B1-B13) II (A18-A21, B14-B21), III (B22-B30), IV (A1-A4). A small amount of intermediate fragment I + IV might be present due to slow hydrolysis of A4-A5 bond. When the peptide maps of insulin lispro and derivative 1 were compared (see Fig. 5) the difference in the retention time of the peak corresponding to fragment III of the derivative and insulin lispro was observed. It was indicative of a modification within fragment III (peptide B22-B31).

**Fig. 6:**
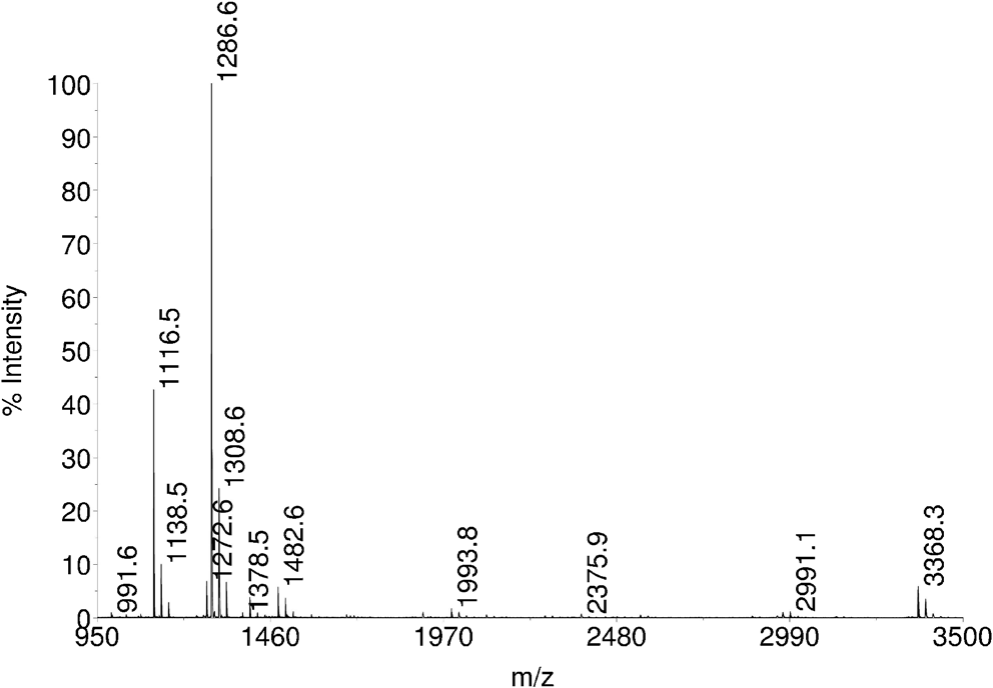
MALDI TOF spectrum of derivative 1 after enzymatic digestion with endoproteinase Glu-C.

Expected peptide fragments resulting from digestion of the derivative with endoproteinase Glu-C are listed in Table I. MALDI analysis of the digest gave a distinct signal at m/z 1286.7 (a sum of 1244.7 + 42; see Fig. 6) which was assigned to peptide B22-B31 (fragment III of the derivative). As this peptide does not contain any serine, further investigation was narrowed down to threonine and lysine residues.

**Table I:**
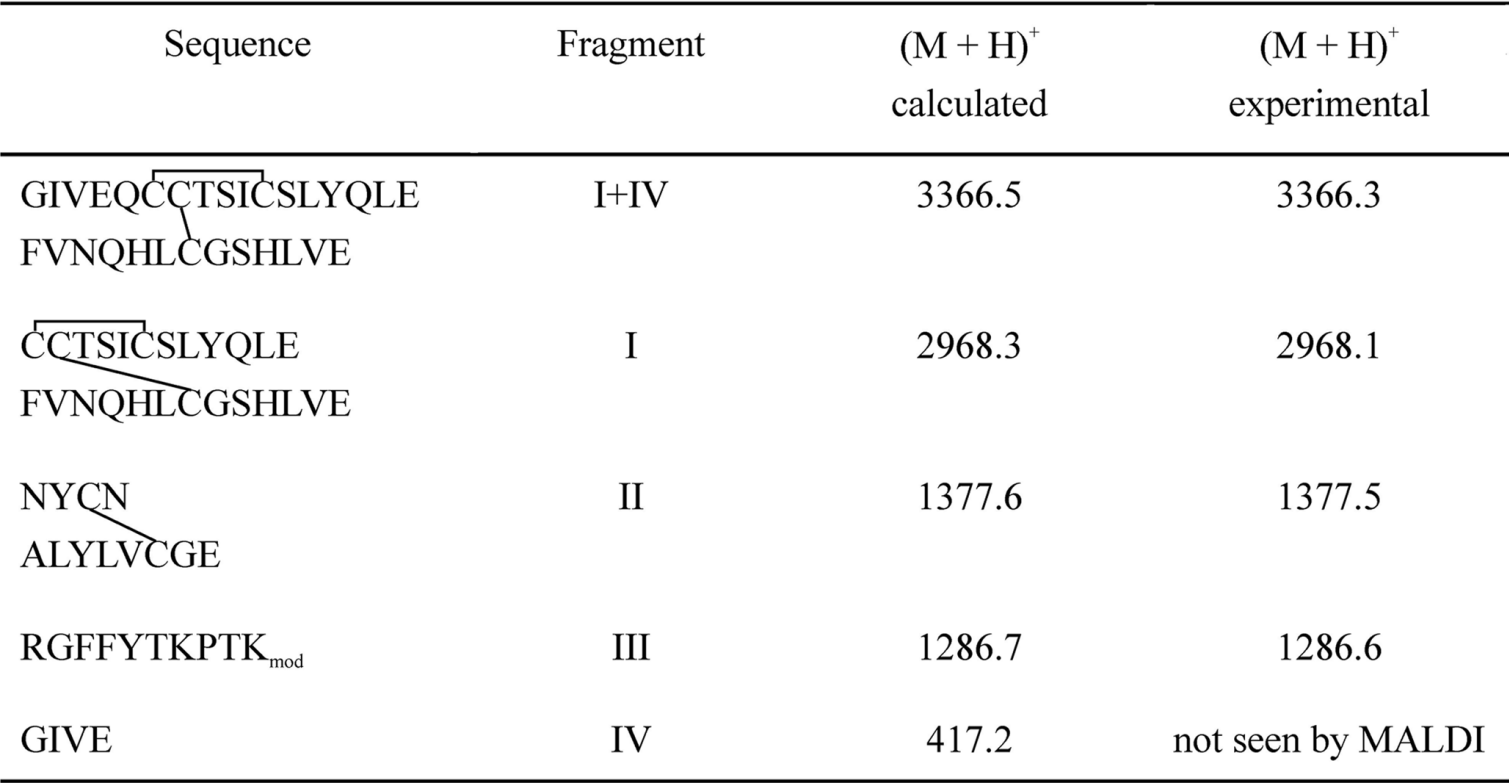
A Comparison Between the Calculated and Experimental Parent Ion Masses (Monoisotopic) of Peptide Fragments Resulted from Digestion of Derivative 1 with Endoproteinase Glu-C

**Fig. 7:**
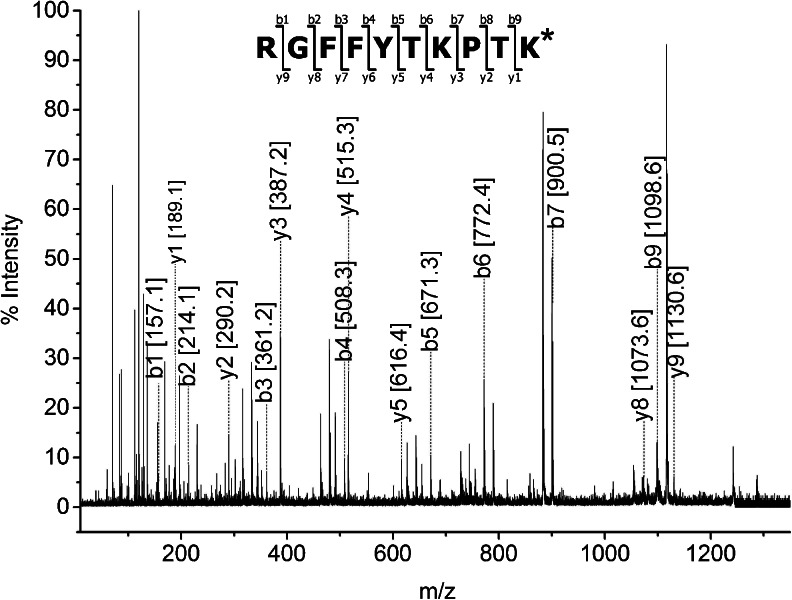
MALDI-TOF/TOF spectrum of peptide B22-B31.

**Fig. 8:**
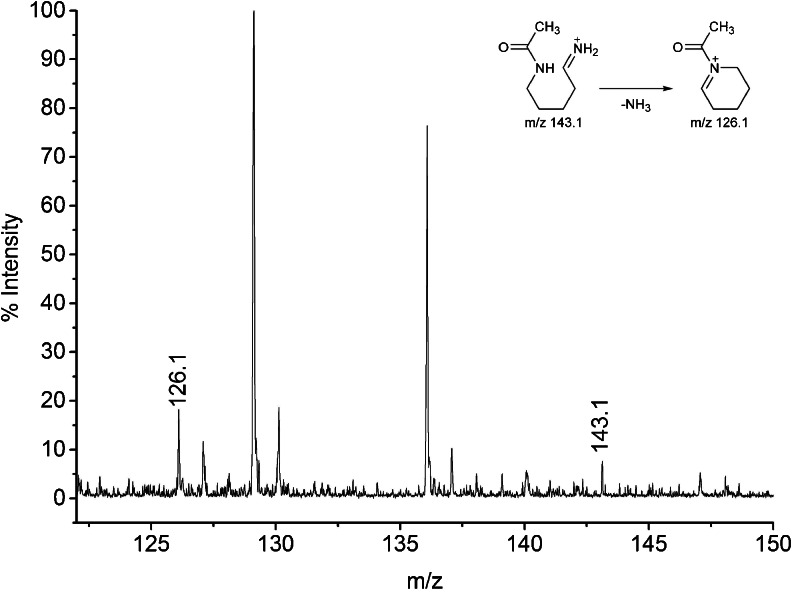
Enlarged MALDI-TOF/TOF spectrum of peptide B22-B31 with diagnostic ions 126.1 and 143.1. Insert – structure of diagnostic ions.

Tandem mass spectrometry was performed on this peptide to explore the location and type of the modification. All b-type ions seen in MS/MS spectrum (Fig. 7) were in agreement with theoretical values, whereas y-type ions were 42 amu over the predicted masses. The presence of two ions: b9 [1098.6] and y1 [189.1] confirms that B30 Thr remains unmodified, while B31 Lys is altered. The sequence of the peptide B22-B31 was as follows: RGFFYTKPTKmod. Closer inspection of the MS/MS spectrum revealed the presence of two diagnostic ions for acetylated lysine at m/z 143.1 and m/z 126.1 (Fig. 8). Although the signal at m/z 143.1 can be also associated with mono-ethylated arginine, the co-presence of 143.1 with 126.1 undoubtedly indicates acetylation. The immonium ion at m/z 126.1 is specific for lysine acetylation and enables discrimination between acetylation and trimethylation of lysine (38).

**Fig. 9:**
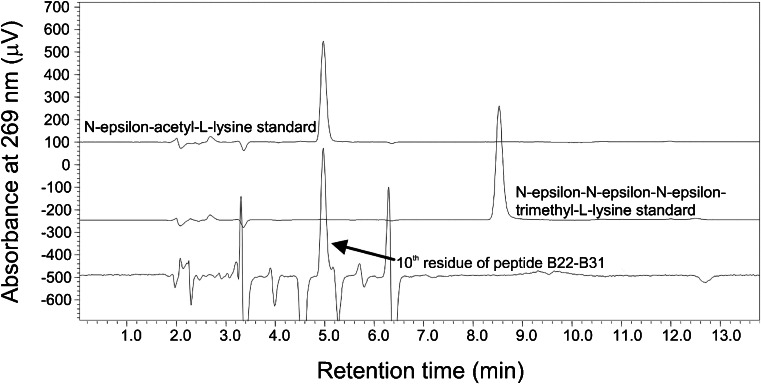
Identification of the 10th residue of the peptide B22-B31 performed during Edman degradation. The illustration presents the HPLC chromatogram of the PTH amino acid residue detached during 10th cycle of sequencing, superimposed on the chromatograms of the acK as well as 3meK standards. The retention time of the 10th residue is identical as that of N-ε-acetyl-L-lysine.

In order to confirm the type of modification in derivative 1, Edman degradation was employed as an orthogonal method. During Edman degradation the automatic protein sequencer consecutively detaches amino acid residues from N-termini of the analyzed polypeptides, converts them into PTH derivatives and performs their identification by HPLC chromatography. Since standards of PTH derivatives of N-ε-acetyl-L-lysine and N-ε-trimethyl-L-lysine are not commercially available, they were synthesized in the laboratory. Analysis of the peptide B22-B31 gave the following 9-mer sequence: RGFFYTKPTKac (N-ε-acetyl-L-lysine was ascertained at 10th position). The chromatograms illustrating the mentioned 10th amino acid residue of this peptide superimposed on acK as well as 3meK standards are presented in Fig. 9. Peaks belonging to the standards of N-ε-acetyl-L-lysine and N-ε-trimethyl-L-lysine differ in retention time sufficiently to allow the exclusion of trimethylation at B31 in the derivative.

On the basis of the presented results it is interesting that a derivative with acetylated lysine at position B28 was dot detected so far. It is possible that acetylation does not take place in other locations than B31 or acetylated derivatives are formed at undetectable levels. A new project is open now in our institute to understand mechanism of acetylation in insulin analogues produced in *E. coli* and to examine all possible acetylation sites. Details of this work will be published in the future.

## CONCLUSIONS

Ion exchange chromatography analysis of related proteins in development batches of recombinant insulin lispro revealed the existence of unknown derivative in excess of the assumed limit 0.1%. This derivative was monitored in all development batches of insulin lispro and it occurred in a range of <LOD – 0.8% depending on purification methods. The molecular mass of the derivative was 42 Da higher that the theoretical mass of Lys(B31) insulin lispro—one of the expected process-related intermediates. Both, Edman sequencing and MS/MS fragmentation provided clear-cut evidence of lysine acetylation and exclusion of trimethylation contingency. The derivative was identified to be N-ε-acetyl-L-lysine (B31) insulin lispro. We do not know at present if acetylation of this insulin lispro derivative proceeds enzymatically or with the participation of acetyl phosphate (AcP) as the acetyl donor, but certainly our discovery is another indication that lysine acetylation is a frequently occurring posttranslational modification in *E. coli*. It is highly probable that our finding does not close the above list of acetylated therapeutic proteins produced in *E. coli.* A worldwide intensification of research on protein acetylation should bring new discoveries in this field.

## ABBREVIATIONS

3meK: N-ε-N-ε-N-ε-trimethyl-L-lysine
acK: N-ε-acetyl-L-lysine
AckA: Acetate kinase
ACN: Acetonitrile
AcP: Acetyl phosphate
IEC: Ion exchange chromatography
KATs: Lysine acetyltransferases
KDACs: Lysine deacetylases
Pta: Phosphotransacetylase
PTC: Phenylthiocarbamyl
PTH: Phenylthiohydantoin
PTM: Post-translational modification
RANTES: Regulated on activation, normal T cell expressed and secreted chemokine
TFA: Trifluoroacetic acid
